# Default mode network mediates low‐frequency fluctuations in brain activity and behavior during sustained attention

**DOI:** 10.1002/hbm.26024

**Published:** 2022-07-29

**Authors:** Hang Zhang, Shi‐You Yang, Yang Qiao, Qiu Ge, Yi‐Yuan Tang, Georg Northoff, Yu‐Feng Zang

**Affiliations:** ^1^ Centre for Cognition and Brain Disorders The Affiliated Hospital of Hangzhou Normal University Hangzhou Zhejiang China; ^2^ Institute of Psychological Science Hangzhou Normal University Hangzhou Zhejiang China; ^3^ Zhejiang Key Laboratory for Research in Assessment of Cognitive Impairment Hangzhou Zhejiang China; ^4^ College of Health Solutions Arizona State University Tempe Arizona USA; ^5^ Institute of Mental Health Research University of Ottawa Ottawa Canada

**Keywords:** default mode network, low‐frequency fluctuation, fMRI, sustained attention

## Abstract

The low‐frequency (<0.1 Hz) fluctuation in sustained attention attracts enormous interest in cognitive neuroscience and clinical research since it always leads to cognitive and behavioral lapses. What is the source of the spontaneous fluctuation in sustained attention in neural activity, and how does the neural fluctuation relate to behavioral fluctuation? Here, we address these questions by collecting and analyzing two independent fMRI and behavior datasets. We show that the neural (fMRI) fluctuation in a key brain network, the default‐mode network (DMN), mediate behavioral (reaction time) fluctuation during sustained attention. DMN shows the increased amplitude of fluctuation, which correlates with the behavioral fluctuation in a similar frequency range (0.01–0.1 Hz) but not in the lower (<0.01 Hz) or higher (>0.1 Hz) frequency range. This was observed during both auditory and visual sustained attention and was replicable across independent datasets. These results provide a novel insight into the neural source of attention‐fluctuation and extend the former concept that DMN was deactivated in cognitive tasks. More generally, our findings highlight the temporal dynamic of the brain–behavior relationship.

## Graphical abstract

DMN shows the increased amplitude of fluctuation, which correlates with the behavioral fluctuation only in the frequency range of 0.01–0.1 Hz. This mediation effect of DMN was observed during both auditory and visual sustained attention and could be replicated across independent datasets. These results provide a novel insight into the neural source of attention‐fluctuation and highlight the temporal dynamic of the brain–behavior relationship.

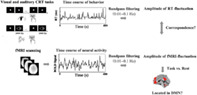

## 1 INTRODUCTION

Sustained attention is critical to maintaining performance over time in a wide range of our daily activities. This is complicated by the fact that our attention spontaneously varies from moment‐to‐moment resulting in fluctuation of behavioral performance (Fortenbaugh et al., 2017; Posner, 2008). Larger fluctuation of attention may lead to severe consequences, such as traffic accidents and medical negligence. Moreover, abnormal fluctuations in attention can be observed in psychiatric and neurological disorders, for example, attention deficit hyperactivity disorder (ADHD; Barkley, 1997). Are their neural fluctuations and how do they relate to the fluctuations in behavior? Addressing these yet unresolved questions is the goal of our study.

Growing studies indicate that sustained attention is a fundamentally rhythmic process (Helfrich et al., 2018; VanRullen, 2018; Zalta et al., 2020). Rhythmic fluctuations in sustained attention have been intensively highlighted in especially the low‐frequency range (Adamo et al., 2014; Di Martino et al., 2008; Sonuga‐Barke & Castellanos, 2007; Yordanova et al., 2011). During the Continuous Performance Test (CPT), subjects show reaction time (RT) fluctuations in the frequency range below 0.1 Hz. The amplitude of this low‐frequency RT‐fluctuation is abnormally increased in participants suffering from attention deficits (Castellanos et al., 2005; Helps et al., 2011). While these RT‐fluctuations reflect attention fluctuation in behavior, their neural correlates remain yet unclear. Is the low‐frequency nature of RT‐fluctuation related to corresponding low‐frequency fluctuation in the brain's neural activity? This is the question guiding our investigation.

The neural basis of sustained attention has been probed in functional magnetic resonance imagining (fMRI) studies using measures of regional activation (Esterman et al., 2013; Langner & Eickhoff, 2013) and functional connectivity (FC; Kucyi et al., 2017; Rosenberg et al., 2016). Despite the different measures, these studies highlight two key networks, namely the attention network (AN) and the default mode network (DMN) in mediating sustained attention. The AN includes the dorsal and ventral frontal–parietal areas, as referred to dorsal attention network (DAN) and ventral attention network (VAN) (Fox et al., 2006). As in its name, the AN shows activity increases from baseline (rest) to attention tasks, that is, activation (Lanssens et al., 2020).

Unlike AN, the DMN's activity exhibits task‐independent decreases in its magnitude, that is, deactivation (Anticevic et al., 2012; Yeo et al., 2011) during attention tasks. This leaves open the exact role of the DMN in sustained attention and especially its fluctuations in the low‐frequency range. Recent investigations show that the magnitude of DMN's activity is correlated with changes in behavior over time (Esterman et al., 2013; Kucyi et al., 2016; Kucyi et al., 2017). Given that the DMN during rest displays strong fluctuations as observed in both low‐frequency range (Fox & Raichle, 2007; Golesorkhi et al., 2020; Raichle et al., 2001) and long timescales (Golesorkhi et al., 2021; Ito et al., 2020; Raut et al., 2020), it may be considered a suitable neural candidate for the low‐frequency fluctuations in sustained attention. Probing the role of DMN in the fluctuation of sustained attention by investigating its neural fluctuation (fMRI) is the main objective of our study.

The DMN does show strong low‐frequency fluctuations in exactly the frequency range of RT‐fluctuation (0.01–0.1 Hz) during attention tasks (Fox & Raichle, 2007; Golesorkhi et al., 2020; Raichle et al., 2001). We, therefore hypothesize that the low‐frequency fluctuations in DMN are related to corresponding low‐frequency fluctuations (0.01–0.1 Hz) in behavior, that is, RT, during sustained attention. Moreover, given the transmodal nature of DMN (Margulies et al., 2016; Northoff et al., 2020; Raichle et al., 2001), we expect that this relationship holds in different sensory modalities like audition and vision. More generally, we assume that low‐frequency fluctuations are shared by both neural and attentional activity as their “common currency” (Northoff et al., 2019, 2020), which is presumed to hold in a transmodal way.

To test our hypotheses, we collected two independent fMRI data sets (one for exploratory and one for replication) during both rest and task states. The task states included a typical CPT of sustained attention, namely two‐choice reaction time (CRT) task (Gomes et al., 2008; Helps et al., 2010). To probe transmodal effects, we applied both auditory and visual versions of the CRT task. RT/fMRI‐fluctuations were assessed across all subsequent trials thus we conduct a more continuous (rather than discontinuous) analysis (Huk et al., 2018). A novel approach to analyzing the brain–behavior relationship is warranted. Both fMRI activity and behavioral performance shall be analyzed in terms of their fluctuations over time (Northoff et al., 2019, 2020) rather than by analyzing fMRI activity in terms of its magnitude (where changes in activity over time are averaged across trials with fluctuations often being discarded as mere noise (Starck et al., 2010). We band‐passed the RT data in the same way as the fMRI data (0.01–0.1 Hz) and track their fluctuations using the same measure, percent amplitude of fluctuation (PerAF) (Jia et al., 2020). The PerAF is a derivate of the well‐known measure of the amplitude of low‐frequency fluctuation (ALFF) but has better reliability (Zhao et al., 2018). These analyses allow us to probe brain–behavior correspondence in the frequency range that is known to exhibit low‐frequency attention fluctuation (Adamo et al., 2014; Di Martino et al., 2008; Sonuga‐Barke & Castellanos, 2007; Yordanova et al., 2011).

## 2 RESULTS

**FIGURE 1:**
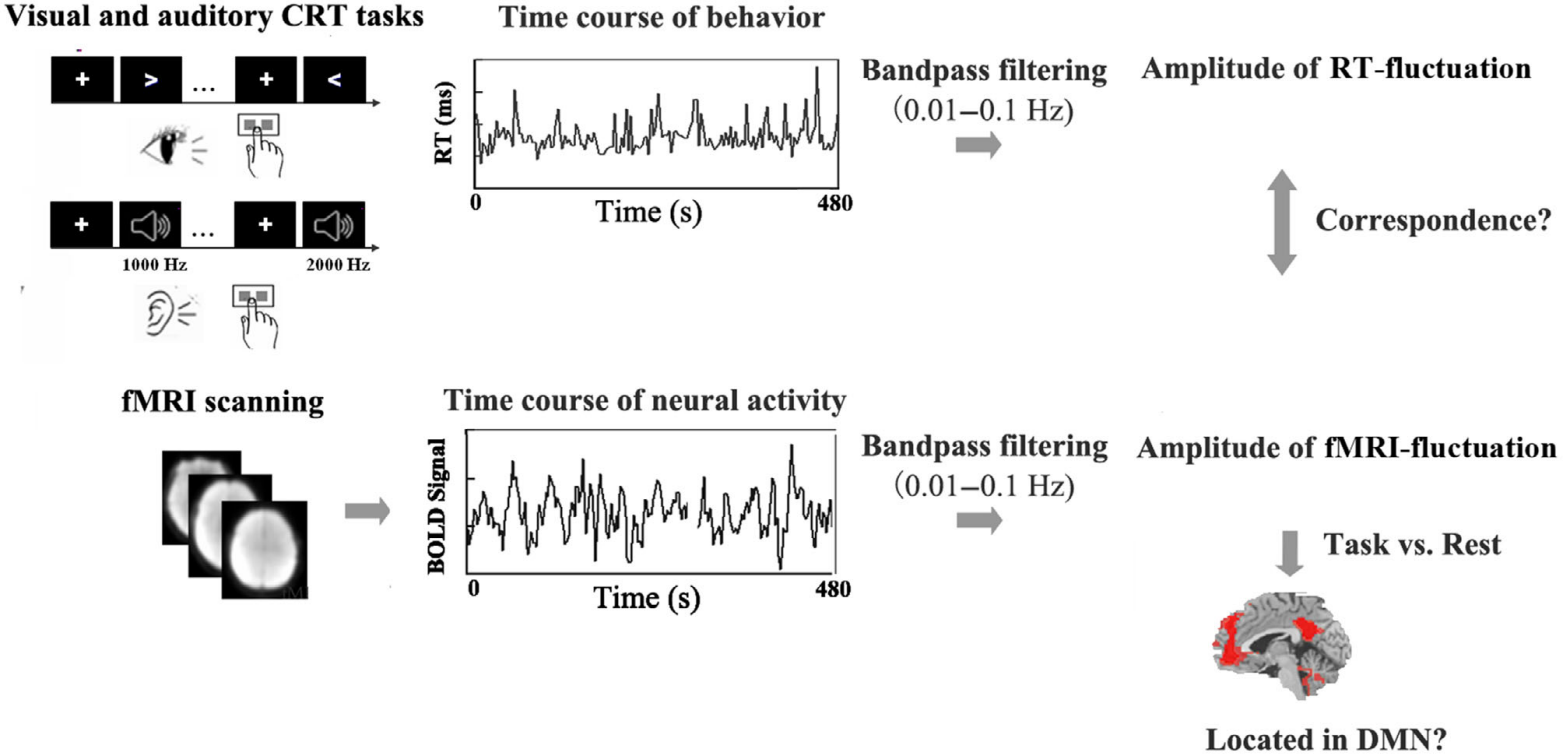
Overview of the methods to characterize the low‐frequency fluctuation of RT and fMRI data. Visual and auditory tasks were employed to conduct transmodal explorations. These analyses were performed on two independent datasets with one serving as testing and the other for replication.

We applied visual and auditory versions of the CRT task for probing sustained attention (Gomes et al., 2008; Helps et al., 2010; Figure 1). This allowed us to explore transmodal similarities in both the behavioral fluctuations and fMRI fluctuations during sustained attention. Subjects performed the visual and auditory tasks while being scanned in fMRI preceded or followed by a resting‐state fMRI (RS‐fMRI) session with a counterbalance sequence (see details in Methods). Since our focus was on the recording and linking low‐frequency fluctuations in the brain (fMRI) and behavior (RT), we applied the same band‐pass filtering in our analysis of both fMRI and RT data, that is, 0.01–0.1 Hz (Figure 1). This allowed us to search fluctuations in both brain and behavior within the same frequency range (serving as their “common currency”; Northoff et al., 2019, 2020). We here include two independent‐samples of the fMRI dataset with one serving as a testing dataset and the other as a replication dataset (see Appendix S1). The paradigm structures of the two datasets are slightly different, that is, interleaving of rest and task blocks for the replication dataset and having them separate for testing dataset (see Methods and Figure S1a); this controls for potential entrainment effects (Huk et al., 2018; Lakatos et al., 2013; Lakatos et al., 2019) in specifically the low‐frequency range (0.01–0.1 Hz) as the target frequency in our study.

### 2.1 Low‐frequency fluctuation in reaction time and transmodal correlation

**FIGURE 2:**
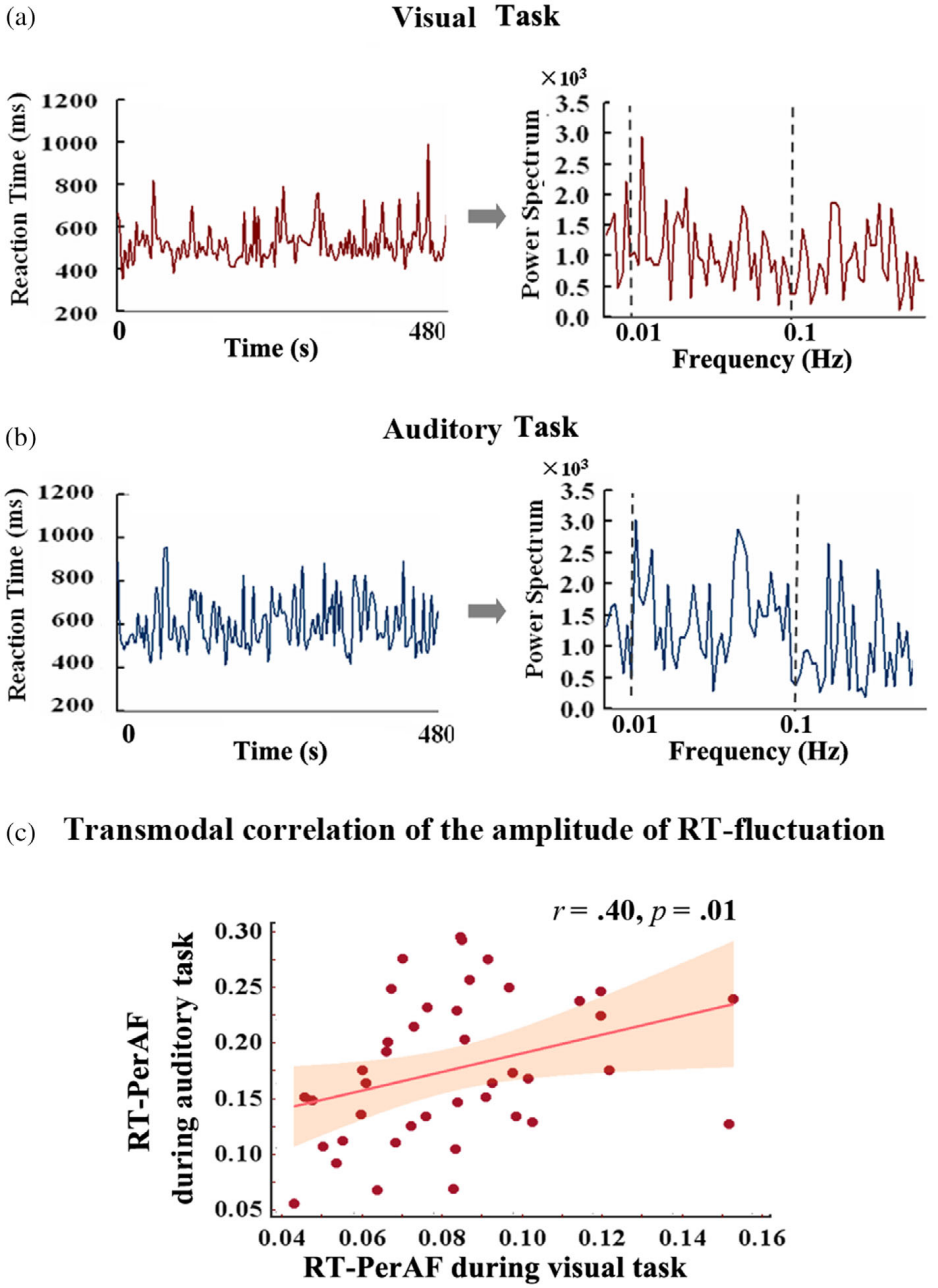
Low‐frequency fluctuation of RT during visual and auditory attention. RT‐fluctuation in visual CRT task (a) and auditory CRT task (b) including their correlation (c) in the low‐frequency range of 0.01–0.1 Hz. The time course of RT was acquired from the subsequent trials during the tasks in visual modality (a) and auditory modality (b). The power spectrum was derived from the RT time course, and the amplitude of RT‐fluctuation (c) was obtained for the low‐frequency range of 0.01–0.1 Hz. No outliers (individual's score >3SD from the group mean) were detected.

We first analyzed the behavioral data (RT and error rate) derived from the visual and auditory tasks (Table S1, see details in Methods). As expected, the RT of both tasks showed relatively high fluctuation in the low‐frequency range of 0.01–0.1 Hz (Figure 2a,b). To probe for transmodal similarity in the RT‐fluctuation, we correlated the PerAF of RT (RT‐PerAF) of auditory and visual tasks. Significant transmodal correlation was determined in the frequency range of 0.01–0.1 Hz (Figure 2c, *r* = .40, corrected *p* < .05) whereas no significant correlation was observed in both slower (<0.01 Hz) and faster (>0.1 Hz) frequency ranges (below 0.01 Hz, *r* = .31, corrected *p* > .05; above 0.1 Hz, *r* = .19, corrected *p* > .05). Further, RT‐PerAF was not correlated with the error rate in each modality (visual task, *r* = .27, *p* = .09; auditory task, *r* = .05, *p* = .75).

Next, we confirmed that the replication dataset yielded similar results with again a significant correlation of auditory and visual RT‐PerAF in 0.01–0.1 Hz (*r* = .44; corrected *p* < .05; see details in Appendix S1 and Figure S1b). Given that the temporal structures of the task paradigm are slightly different between the testing and replication datasets, we probed the difference of the RT‐PerAF between the two datasets. The transmodal correlation of RT‐PerAF shows no significant difference between testing and replication (*r* = .40 for testing vs. *r* = .44 for replication, *z* = −.20, *p* > .05; see details in Appendix S1 and Table S1). This suggests that the transmodal correlation of RT‐PerAF is not related to the temporal structure of the paradigm (as that was slightly different between testing and replication data sets).

Together, our behavioral data show clear low‐frequency fluctuations in RT (0.01–0.1 Hz) in both auditory and visual versions of the CRT task. The amplitude of the low‐frequency fluctuations in both auditory and visual RT was correlated with each other suggesting a transmodal effect.

### 2.2 Activation and fluctuation during sustained attention recruiting distinct networks

We next probed both task‐related magnitude and fluctuation of neural activity during the CRT. For that purpose, in addition to the typically measured activation, that is, increased magnitude of activity from rest to task (Esterman et al., 2013; Langner & Eickhoff, 2013), we also examined the amplitude of intra‐regional fluctuation over time using the measure of PerAF (Jia et al., 2020; Zhao et al., 2018). This allowed us to determine which regions exhibit task‐related activation, that is, the magnitude of activity, and which ones show task‐related changes in their fluctuations, that is, variance in their magnitudes over time.

**FIGURE 3:**
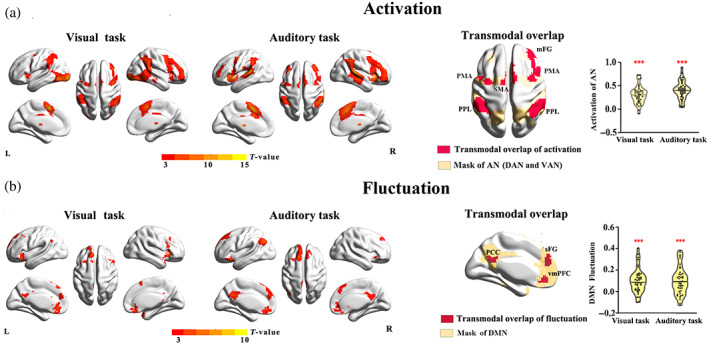
Activation and fluctuation of fMRI activity during visual and auditory attention. Activation for both visual and auditory modalities and transmodal overlap (a). The overlapping regions are mainly located in the mask of the attention network (combined masks of dorsal attention network and ventral attention network). Fluctuations during both visual and auditory tasks and transmodal overlap (b). The transmodal overlap of fluctuations is mainly located in the mask of the default mode network. In (a) and (b), the network masks were released by Yeo et al. (2011), and activation (task > rest) and fluctuation (task < rest) were corrected with FDR, *q* < 0.05, cluster size >10 voxels. Activation was assessed using GLM to generate task‐rest contrasts of the beta value. Amplitude of fluctuation was measured with the task‐rest difference of PerAF in 0.01–0.1 Hz. Violin plots indicate activation of AN (beta value) and task‐related fluctuation of DMN (task‐rest difference of PerAF value in 0.01–0.1 Hz) in both visual and auditory tasks. Solid line in the plots indicates the median value across all participants, and dot markers indicate the individual data. Asterisks represent the level of significance of one sample test, *<.05,**<.005, ***<.001 (all corrected for multiple comparison).

We found that regions distributed in the frontal–parietal areas, insula, thalamus, and cerebellum exhibit activation during CRT tasks in both auditory and visual modalities (Figure 3a and Table S2). Most of these regions are located in the dorsal and ventral AN (DAN and VAN) defined by previous publications (Scheibner et al., 2017). Of note, some of the regions showing activations in the VAN, that is, insula and ventral frontal areas are also associated with the salience network (SN) (Menon & Uddin, 2010; Scheibner et al., 2017).

In contrast to the activation pattern recruiting mainly AN regions, the analyses of task‐related fluctuation yielded a different pattern. Both visual and auditory CRT tasks showed increased PerAF in the DMN regions but not in AN (Figure 3b and Table S2). We further confirmed that the activation and fluctuation recruit different brain networks, that is, concerning for DMN (fluctuation) and AN (activation) (Figure S2a). Moreover, rather than activation, as in AN, the DMN was deactivated during both visual and auditory CRT tasks (Figure S3). Interestingly, the core midline DMN regions showing strong deactivation, that is, posterior cingulate cortex (PCC) and ventral medial prefrontal cortex (vmPFC), also displayed strong task‐related fluctuations (Figure S2b).

**FIGURE 4:**
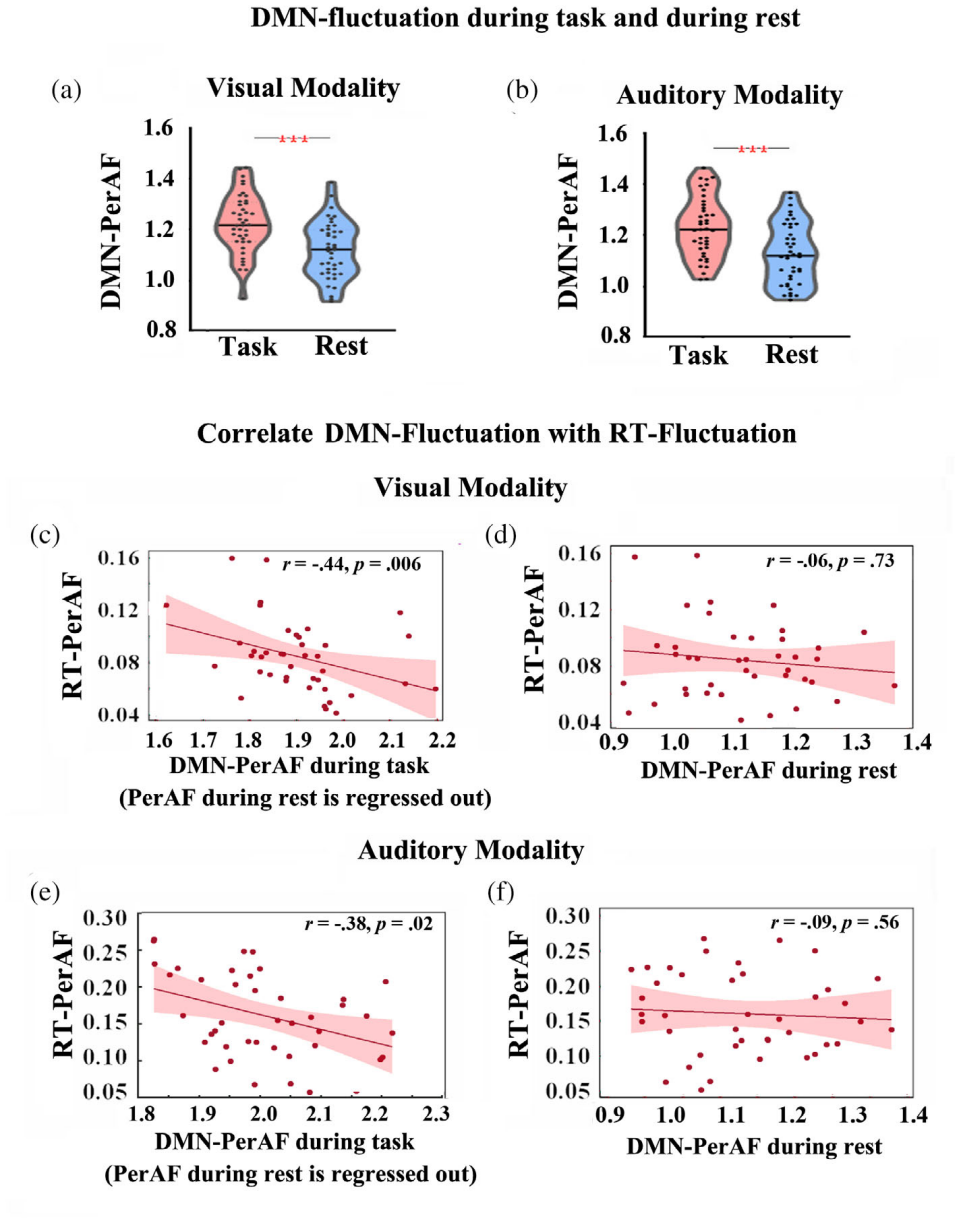
The amplitude of DMN‐fluctuation during task and rest, and its correlation with the amplitude of RT‐fluctuation. Significant task‐rest difference of the amplitude of DMN‐fluctuation during visual (a) and auditory (b) tasks. Violin plots indicate the amplitude of fluctuation (PerAF value) of DMN in both visual and auditory modalities. Solid line in the plots indicates the median value across all participants, and dot markers indicate individual data. The amplitude of RT‐fluctuation shows significant correlation with the amplitude of DMN‐fluctuation during both visual task (c) and auditory task (e). These correlations were not obtained during its rest state (d) and (f). No outliers (individual's value >3SD from the group mean) were detected. Task‐rest differences of the amplitude of DMN‐fluctuation in 0.01–0.1 Hz were examined at group level with paired t test. Asterisk represents the level of significance, *<.05,**<.005, ***<.001 (all corrected for multiple comparison).

Finally, we replicated these findings in our second dataset. Despite the modified temporal structure of the paradigm in the replication data set (having rest and task separate rather than interleaving rest and task blocks, see details in Methods), the DMN again showed a task‐related increase of PerAF in the paradigm and predominant deactivation in especially the midline regions. This suggests that the rest‐task changes in neural fluctuations of DMN are not dependent upon the temporal structure of the paradigm but rather on the task itself including its attention fluctuations (as it will be probed in the next steps; Figure 4a,b).

### 2.3 Brain–behavior relationship: DMN‐fluctuation correlates with RT‐fluctuation

We next investigate the relationship between DMN‐fluctuation and RT‐fluctuation. We observed that the DMN‐PerAF during CRT tasks showed a negative correlation with RT‐PerAF: the higher and stronger the task‐related changes in the neural fluctuations of the DMN, the lower the fluctuations in the RT. This again was observed in both modalities, that is, visual (*r* = .44, *p* = .006) and auditory (*r* = .38, *p* = .02) tasks, as well as in different temporally structured paradigms, that is, separate rest and task states versus interleaving rest and task blocks (Figures 4c,e and S5). We further confirmed that this correlation could not be determined beyond the frequency range of 0.01–0.1 Hz (each |*r*| < .15, each *p* > .05; Figure S4). We also explored this correlation in some specific frequency bands, that is, Slow‐5 (0.01–0.027 Hz) and Slow‐4 (0.027–0.073 Hz), in accordance with previous studies (Di Martino et al., 2008; Helps et al., 2011). Correlation between DMN‐fluctuation and RT‐fluctuation in the two specific frequency bands was significant for visual modality (in Slow‐5, *r* = −.35, *p* = .027; in Slow‐4, *r* = −.43, *p* = .007) but was not significant for auditory modality (in Slow‐5, *r* = .02, *p* = .92; in Slow‐4, *r* = −.29, *p* = .06). Thus, sub‐frequency bands could not directly contribute to the trans‐modal correspondence between DMN‐fluctuation and RT‐fluctuation in low‐frequency range.

Moreover, we excluded carry‐over effects from the DMN‐fluctuation in the resting state as the DMN‐PerAF in the resting state did not show correlation with RT‐PerAF (each |*r*| < .09, each *p* > .05, Figure 4d,f). Given that some additional factors, for example, age and gender potentially affect the behavioral performance of sustained attention (Bielak et al., 2014; Blatter et al., 2006), we also control for these factors in the brain–behavior correlation analysis. Covarying the age and gender of participants, the DMN‐RT correlations were preserved for both modalities (visual task, *r* = −.41, *p* = .009; auditory, *r* = −.35, *p* = .03) (see Discussion for unraveling the seemingly counterintuitive nature of the negative [rather than positive] correlation of neural and behavioral fluctuations).

### 2.4 DMN FC‐fluctuation versus DMN‐amplitude fluctuation

The neural fluctuations characterized by PerAF reflect the amplitude of intra‐regional fluctuations. To rule out the potential impact of inter‐regional fluctuations, we also examined fluctuations of functional connectivity (FC) between regions, that is, dynamic FC as to investigate whether FC‐fluctuations are related to RT‐fluctuation (see details in Methods and Appendix S1).

The engagement of the AN and DMN in sustained attention has been related to the inter‐regional functional connections within DMN and AN (intra‐network FC) as well as the ones between DMN and AN (inter‐network FC) (Bonnelle et al., 2011; Huang et al., 2020; Zhang et al., 2015). Given that the low‐frequency fluctuations were significantly increased in the DMN but not in the AN during sustained attention, we examined whether the FC‐fluctuations within these networks could account for this difference. Here, we did not find the difference between DMN and AN in their intra‐network fluctuations during tasks (see details in Methods; Figure S6).

Importantly, neither intra‐network fluctuations nor inter‐network fluctuations of FC showed a significant correlation with RT‐fluctuations (Table S5, each |*r*| < .3, each *p* > .05). Next, we tested the strength of FC between DMN and AN (Kelly et al., 2008; Zhang et al., 2015). The strength of FC between AN and DMN did not exhibit a significant correlation with RT‐fluctuations during visual and auditory tasks (Table S5). Together, these findings indicate that fluctuations in inter‐regional FC can neither account for the DMN‐AN difference concerning intra‐regional fluctuation and activation nor the relationship of DMN‐fluctuation with RT‐fluctuation.

## 3 DISCUSSION

The goal of our study was to investigate whether fluctuations in sustained attention are related to neural fluctuations in the same low‐frequency range. We show that intra‐regional neural fluctuations in the low‐frequency range (0.01–0.1 Hz) of the DMN are related to attention fluctuations in the same frequency range. While the AN shows activation (the magnitude of activity rather than fluctuation) during the same attention task. This suggests the “work sharing” of neural networks with respect to fluctuation (DMN) and activity magnitude (AN) during sustained attention. Together, we here for the first time unravel a neural source of attention fluctuation by identifying neural fluctuation of DMN in a corresponding low‐frequency range. Beyond showing the temporal dynamic of brain–behavior relation for sustained attention, our findings shed a novel light on the role of the DMN in behavior and sustained attention.

### 3.1 DMN‐fluctuation versus AN‐activation

The main finding of our study is the involvement of different brain networks in neural fluctuation and activation. The AN (including both DAN and VAN) shows a task‐related increase in activity magnitude during both auditory and visual sustained attention tasks whereas no change in AN fluctuation was observed. In contrast, DMN fluctuations significantly increased from rest to task while the activation magnitude was not elevated. Increase in fluctuations concerned specifically intra‐regional activity, that is, the variation in the amplitude over time as measured by PerAF.

Our findings add to the distinction of DMN and AN in their roles in attention. The AN is related to externally‐oriented attention and cognition while the DMN mediates more internally‐oriented processes (Christoff et al., 2016; Corbetta et al., 2008; Huang et al., 2020). That is mainly derived from the magnitude of their activation in attention tasks (Esterman et al., 2013; Kucyi et al., 2016). Nevertheless, unlike the activated AN, the DMN is usually inactive in tasks—this leaves open the contribution of DMN to especially sustained attention. Our findings address this gap by showing that DMN and AN are involved in distinct ways in sustained attention, namely through activation (AN) and fluctuation (DMN). Both networks thus contribute distinct aspects to the processing of sustained attention—they “share the work.”

Physiologically, the DMN is characterized by strong slow activity fluctuations in the resting state as observed in both frequencies (Fransson, 2005; Golesorkhi et al., 2020) and the time domain (Golesorkhi et al., 2021; Ito et al., 2020; Raut et al., 2020). The behavioral or cognitive role of DMN infra‐low frequency fluctuations remains somewhat unclear, though. Various studies associate the DMN with different forms of internally‐oriented cognition like mind‐wandering (Christoff et al., 2016; Northoff, 2018), mental time travel (Northoff & Huang, 2017; Schacter et al., 2012), and self‐referential cognition (Northoff, 2016, 2006; Wen et al., 2020). Here we extend these observations by showing that the DMN and specifically its intra‐regional low‐frequency fluctuations are associated with the behavioral fluctuations in sustained attention, that is, CRT task. Given that the CRT task is a form of externally‐oriented cognition, our findings add to the growing evidence that DMN is not only involved in internally‐oriented cognition but also in externally‐oriented cognition (Buckner & DiNicola, 2019; Yeshurun et al., 2021).

### 3.2 Correlation of DMN‐fluctuation with RT‐fluctuation

The involvement of DMN fluctuation in sustained attention is further supported by its correlation with RT‐fluctuation during the CRT tasks. Specifically, task‐related fluctuation of DMN (as distinguished from the resting state fluctuations which were regressed) correlated negatively with the RT fluctuation during the CRT task: the more task‐related neural fluctuation in DMN, the less behavioral fluctuation in RT during sustained attention. This relationship holds specifically for RT‐fluctuation but not for error, that is, accuracy. While low‐frequency neural fluctuations are well known in DMN (Fox & Raichle, 2007; Golesorkhi et al., 2021; Raichle et al., 2001; Raut et al., 2020), their roles in cognition remain yet unclear. Our negative DMN‐CRT correlation suggests that low‐frequency neural fluctuation in DMN contributes to containing behavioral fluctuation during sustained attention.

At first glance, the negative direction of the correlation appears paradoxical as more DMN fluctuation is related to less RT‐fluctuation. However, one needs to consider this result together with the physiology of DMN fluctuation. The DMN exhibits strong power in the low‐frequency range (0.01–0.1 Hz) whose fluctuations are characterized by long cycle durations (Fox & Raichle, 2007; Golesorkhi et al., 2020). These long cycle durations are ideally suited for integrating different time points within DMN neural activity, i.e. temporal integration (He & Raichle, 2009; Northoff & Huang, 2017; Zilio et al., 2021). The DMN's high degree of temporal integration of inputs across different time points, in turn, may allow a more stable and less fluctuating behavioral‐cognitive performance of the CRT task over time hence the negative DMN‐RT correlation. This is further supported by the fact that we applied the same bandpass for both neural and behavioral analysis suggesting their temporal correspondence, that is, “common currency” (Northoff et al., 2019, 2020) of DMN and behavior in the low‐frequency range (0.01–0.1 Hz).

The importance of high degrees of temporal integration is further supported by the long timescales of the DMN (Golesorkhi et al., 2021; Ito et al., 2020; Raut et al., 2020). This allows the DMN to mainly operate in a transmodal way during different sensory modalities (Golesorkhi et al., 2020, 2021; Margulies et al., 2016), that is, by integrating inputs from different sources or origins. The transmodal nature of DMN is further supported by our finding that DMN‐fluctuation mediated both visual and auditory attention tasks including their respective RT‐fluctuation; this supports a common transmodal mechanism of neural fluctuation for facilitating the transfer of information across sensory modalities (Barne et al., 2018; Senkowski et al., 2008). Future imaging and modeling studies are warranted to investigate whether these transmodal fluctuations are related to high degrees of temporal integration enabled by the strong low‐frequencies with their long cycle durations in DMN.

### 3.3 Limitations of the current study

A few limitations of our study were recognized. First, the sampling rate of fMRI was 2 s; this prevented us to explore the neural fluctuation in a higher frequency range (>0.25 Hz). Fast scanning is required to further illustrate whether a neural fluctuation in such a relatively high‐frequency range has a functional role in sustained attention. Second, it is unclear to which degree our results generalize to other tasks. Sustained attention is implicated in almost all cognitive tasks. Here, we used the CRT task as it is quite simple relative to other more complex tasks, for example, gradCPT (Rosenberg et al., 2013), Eriksen Flanker task (Servant & Logan, 2019), etc. Moreover, given that the sampling rate of RT is usually 2–3 s, it only allows us to explore the low fluctuation of behavior while faster RT sampling is required for involving the faster frequency range (>0.25 Hz). Whether fast behavioral fluctuations are related to corresponding neural fluctuation in a common frequency domain remains to be addressed with different paradigms. Finally, it will be critical to determine the clinical significance of our results like for ADHD, where both attention deficits and DMN changes are well known (Sutcubasi et al., 2020).

## 4 CONCLUSION

In summary, the present study suggests that the neural fluctuations of a key network in the brain, the DMN, mediate behavioral fluctuations during sustained attention in a corresponding low‐frequency range (0.01–0.1 Hz). This provides insight into the temporal dynamics of the direct brain–behavior relationship during sustained attention and sheds novel light on, so far, the elusive role of the DMN in cognition and behavior.

## 5 MATERIALS AND METHODS

### 5.1 Participants

A total of 100 healthy participants were recruited as two independent datasets, each *n* = 50 (dataset I:27 females, 22 ± 1 years old; dataset II: 25 females, 21 ± 2 years old). All of the participants were right‐handed, normal hearing and normal or corrected‐to‐normal visual acuity as measured by clinical hearing, and vision tests. No individual reported any history of neurological and psychiatric diseases. Seventeen participants (8 participants of the dataset I and 9 participants of the dataset II) dropped out because of malfunction of the equipment/excessive head motion (head motion >2 mm translation or >2° rotation in any direction). At last, 42 participants in the dataset I (22 females, 22 ± 1 years old) and 41 participants in the dataset II (22 females, 21 ± 1 years old) participants were recriuted in this study. All participants gave written informed consent prior to their participation. The study was approved by the Center for Cognition and Brain Disorders (CCBD) Ethics Committee of Hangzhou Normal University.

### 5.2 Experimental tasks of sustained attention

Two‐choice reaction time (CRT) paradigm was employed to construct visual and auditory tasks of sustained attention since this paradigm is suitable for conducting transmodal exploration and frequency‐dependent analysis of behavior (Gomes et al., 2008; Helps et al., 2010). The auditory task consisted of two sine tones of 1000 and 2000 Hz at a sound pressure level of 90 dB, and the visual CRT contained two visual stimuli, that is, “>” and “<.” Stimuli types occur with equal frequency, as did the target arrows/tones in a randomized order in each scanning run. In each trial of the scanning run, the stimuli were pseudo‐randomly presented for 500 ms interleaved with a fixation cross, and the inter‐trial‐interval (ITI) was 3000 ms. Each participant had to press the button on the response pad with their left/right index finger to determine the sine tones (auditory task) /the direction of the arrow (visual task), and they were instructed to respond as quickly and accurately as possible. Each participant had a 1‐min practice period to get familiar with the related procedure before each task.

The visual and auditory CRT tasks were conducted in a slightly different temporal structure, that is, block structure and state structure, which controls for potential entrainment effects (Huk et al., 2018; Lakatos et al., 2013; Lakatos et al., 2019) in specifically the low‐frequency range (0.01–0.1 Hz) as the target frequency in our study. The block structure interleaves task and rest blocks (40 trials, 2‐min for each), which allows the activation and the low‐frequency fluctuation to be examined simultaneously. In rest blocks, participants were requested to fixate a cross in the middle of the screen. Visual and auditory CRT tasks were conducted in independent runs with counterbalanced order, while each run lasted about 12 min including three task blocks and three rest blocks. A modified structure involves separate task and rest states (160 trials, 8‐min for each). Visual/auditory task and the corresponding resting state were obtained in independent scanning runs. The assignment of modality orders and order of task and rest states were counterbalanced across all participants.

### 5.3 RT‐fluctuation analysis

Low‐frequency fluctuations (<0.1 Hz) have been intensively highlighted in behavioral explorations of sustained attention and attention deficit (Adamo et al., 2014; Di Martino et al., 2008; Sonuga‐Barke & Castellanos, 2007; Yordanova et al., 2011). This low‐frequency pattern has been recognized in the neural activity measure with fMRI (Fox & Raichle, 2007; Golesorkhi et al., 2020; Raichle et al., 2001). These evidences guide us to analyze behavioral and neural fluctuations within the same frequency range, that is, bandpass filtering along 0.01–0.1 Hz and using the same measure, perAF. This methodological operation ensured us to get insight into fluctuations shared by RT and brain providing the “common currency” of brain and behavior (Margulies et al., 2016; Northoff et al., 2020).

Behavioral data (RT and error rate) derived from different paradigm structures were analyzed separately, one serving as a testing (state structure) and the other for replication (block structure). For the testing, the first 6‐s RT data were discarded, as the participants were still accustomed to the task during these trials. Then, missing and anticipatory responses (RT < 100 ms) were interpolated by averaging the two immediate neighboring trial responses to reconstruct an integrated time series of RT (Di Martino et al., 2008; Helps et al., 2011). Band‐filtered (0.01–0.1 Hz) were followed, and the PerAF of the filtered RT data were calculated

$$\mathrm{RT}‐\text{PerAF}=\frac{1}{n}\sum_{i-1}^{n}\left|\frac{\mathrm{Xi}-u}{u}\right|$$

$$\;u=\frac{1}{n}\sum_{i-1}^{n}\mathrm{Xi}$$

where *Xi* is the RT value of the *i*th time points, *n* is the number of time points of a given time series, and *u* is the mean value of RT of that time course. Besides this, error rate (including both omission errors and commission errors) were calculated based on the behavioral data of each task.

We used correlation analysis to test whether the RT‐fluctuation is related to the error rates. Then, we determined the transmodal correlations of the RT‐fluctuation (PerAF value) of both visual and auditory modality in the target frequency (0.01–0.1 HZ) as well as in the frequency ranges below 0.01 Hz and above 0.1 Hz (as to frequency‐specificity). Since the RT‐fluctuations and error rates were not normal distribution. Spearman correlation was employed in these analysis. Bonferroni corrections were applied to each test to adjust for multiple correlations.

As for the replication dataset, RT data were processed using the identical procedures as described above (see details in Appendix S1).

### 5.4 MRI acquisition

Images of all experiments were acquired on a 3 T GE scanner (MR‐750, GE Medical Systems, Milwaukee, Wisconsin) at the center for Cognition and Brain Disorder of Hangzhou Normal University (HZNU). The fMRI and structure image data from Experiments I and II were collected by using the same parameter. Multislice T2*‐weighted fMRI images (repetition time [TR] = 2000 ms; echo time [TE] = 30 ms; flip angle [FA] = 90°; 43 slices with interleaved acquisition; matrix = 64 × 64; field of view [FOV] = 220 mm) and high resolution T1 images (176 sagittal slices, thickness = 1 mm, TR = 8.1 ms, TE = 3.1 ms, FA = 8°, FOV = 250 mm) were acquired in each participant. Data of arterial spin labeling (ASL) were also acquired from the participants of state‐testing, and analysis of these ASL data was not involved in the present study.

During all of the experiments, participants wore MRI‐compatible earphones in combination with earplugs. The auditory stimuli were presented via earphones and the visual stimuli were displayed to the participant via a mirror mounted on the head coil that reflected visually presented instructions on a semi‐transparent screen at the end of the scanner bore. Cushions inside the head coil were used to reduce head movement. The button‐response was performed with the response pad which was connected to a computer running the E‐prime program (Psychology Software Tools, Pennsylvania) to record the responses.

### 5.5 fMRI analysis

We preprocess the fMRI data from each scanning run using identical procedures, and the procedures were implemented in. the DPABI_V2.31 toolbox (Yan et al., 2016) and SPM12 (http://www.fil.ion.ucl.ac.uk/spm/software/spm12). The first three volumes were removed. Slice timing correction, image realignment to correct head motion were followed. After individual structural images were segmented after co‐registered to functional images, functional images were spatially normalized to Montreal Neurological Institute (MNI) space at 3 mm isotropic voxel resolution applying the unified segmentation parameters. The linear trend, head motion parameter measured by Friston‐24 model, white matter (WM), and cerebrospinal fluid (CSF) signals were further regressed out as nuisance covariates. All images were spatially smoothed with a 6 × 6 × 6 full‐width at half maximum (FWHM) Gaussian kernel (Yan et al., 2016).

### 5.6 Neural activation and fluctuation

Neural activity was analyzed in terms of magnitude (activation) and fluctuations. fMRI activation was assessed following the standardly used procedure (Friston et al., 1995). Using the rest blocks as the baseline, a general linear model (GLM) was applied for each subject's data processed by a global scaling with SPM12. Regions showing activation (task > rest) and deactivation (rest > task) were identified with one‐sample *t* test with multiple comparison correction (*q* < 0.05, FDR correction).

To identify the transmodal activation and deactivation, the thresholded t‐maps were binarized and overlapped across visual and auditory modalities. The overlapped voxels were quantified with Dice's coefficient (Nei & Li, 1979).

The fluctuation was recorded across all trials over time, thus conducting a relative continuous analysis (Huk et al., 2018). Here, the fluctuation was tracked with the measure, PerAF, which reflects the intra‐regional amplitude of fluctuations. PerAf was calculated as follows: (Christoff et al., 2016).

$$\;\text{PerAF}=\frac{1}{n}\sum_{i-1}^{n}\left|\frac{\mathrm{Xi}-u}{u}\right|$$

$$u=\frac{1}{n}\sum_{i-1}^{n}\mathrm{Xi}$$

where, *Xi* is the fMRI intensity of the *i*th time points; *n* is the number of time points of a given time series, and *u* is the mean magnitude of that time series. The calculation of PerAF was performed with the toolkit of RESTplus v1.2 (Jia et al., 2019) (http://www.restfmri.net/forum/RESTplusV1.2).

Preprocessed fMRI data of each block was band‐filtered (0.01–0.1 Hz), and then PerAF of each voxel was calculated and normalized by dividing the global mean value. Voxel‐based PerAF was averaged across all task blocks/rest blocks, respectively. Task‐rest differences of PerAF were identified through the paired *t*‐test (task vs. rest) with multiple comparison correction (*q* < 0.05, FDR corrected).

The task‐related increase in the fluctuation of the amplitude was determined using the thresholded t‐maps. Binarized t‐maps were overlapped across visual and auditory modalities. The number of overlapping voxels was quantified with Dice's coefficient (Nei & Li, 1979). These overlapped regions were employed as a whole seed of interest (corresponding to DMN) in the subsequent analysis (the same procedure of prior studies; Kelly et al., 2008; Zhang et al., 2015).

### 5.7 Brain–behavior correlations

DMN‐fluctuations were further tested for the modified task structure of separating task and rest states. PerAF value from the DMN seed was extracted for each participant. Then, the task‐rest difference of DMN‐fluctuation was determined using paired *t*‐test. Next, we examined whether the DMN‐fluctuation during the task is correlated with the RT‐fluctuations in the same frequency range of 0.01–0.1 Hz and whether this correlation results from a carry‐over from the rest to the task. These correlations were performed with Spearman correlation analysis on DMN‐fluctuation during both task and rest (due to non‐normal distribution of the data), and for probing the transmodal similarity, these correlation analyses were also conducted in each visual and auditory modality separately. To rule out the influences from gender or age of the participants, we regressed out these factors to verify the brain–behavior correlations. Furthermore, probing the frequency specificity of the brain–behavior relationship, we also verified these correlations in the frequency ranges below 0.01 Hz and above 0.1 Hz.

### 5.8 Inter‐regional and intra‐regional fluctuation of FC

We next investigated the inter‐regional fluctuation by dynamic FC as distinguished from intra‐regional fluctuation (PerAF analysis). Dynamic FC was assessed in terms of intra‐network and inter‐network manners. For all the seed regions (Table S2 and S3), intra‐network FC and inter‐network FC were computed within sliding windows (a window width size of 30 TRs = 60s and sliding steps = 1 TR) (Allen et al., 2014; Handwerker et al., 2012). FC was assessed in each window by extracting the time courses of the seeds and calculating the Pearson correlation coefficient between each seed pair. The resulting coefficients were Fisher transformed to produce the *z* values. For intra‐network FC, we averaged the *z* value of the FC coefficient of each seed pair to get the mean FC value. For the inter‐network FC, we extracted the time courses from the whole network regions and calculated the Pearson correlation coefficient between networks. Then, we characterized intra‐network/inter‐network fluctuations of FC by calculating the standard deviation of the Fisher's *z*‐transformed Pearson's correlation coefficients across all of the sliding windows for each participant.

These analyses were verified by using a window width size of 15 TRs and 60 TRs in replication. The correlation of intra‐network fluctuation/inter‐network fluctuation of FC with RT‐fluctuation was also evaluated. Motivated by prior works (Huang et al., 2020; Zhang et al., 2015), we also examined the strength of inter‐network FC. The averaged time courses (derived from all TRs of the whole scanning run) from the AN or DMN regions were extracted, and the Pearson correlation coefficient between the time courses of AN and DMN, that is, the strength of inter‐network FC, was calculated. The association of Fisher's *z*‐transformed strength of inter‐network FC with the amplitude of RT‐fluctuation was further evaluated.

## AUTHOR CONTRIBUTIONS

Yu‐Feng Zang and Hang Zhang conceived and designed the experiment. Hang Zhang, Shi‐You Yang, Yang Qiao and Qiu Ge collected the data and performed the data analysis. Yu‐Feng Zang, Georg Northoff and Yi‐Yuan Tang provided advice on the analysis and interpretation of the results. Hang Zhang, Yu‐Feng Zang, and Georg Northoff drafted and revised the manuscript. All authors contributed to the article and approved the submitted version.

## CONFLICT OF INTEREST

The authors declare no conflicts of interest.

## Supporting information


**Appendix S1** Supporting Information.

## Data Availability

The data of this article will be made available by the authors upon request. Requests to access the datasets should be directed to the corresponding author. Human data will become available following the CCBD's regulations.
