# Supplementary material for: Default mode network mediates low‐frequency fluctuations in brain activity and behavior during sustained attention
Source: Hum Brain Mapp. 2022 Jul 29;43(18):5478–89. doi: 10.1002/hbm.26024 (PMC9704793; doi:10.1002/hbm.26024)
Supplement: Supplementary file 1 — Appendix S1 Supporting Information. [file HBM-43-5478-s001.doc]

**Supplementary Information for**

Low-frequency fluctuations connect brain and behavior during sustained attention

Hang Zhang, Shi-You Yang, Yang Qiao, Qiu Ge, Yi-Yuan Tang, Georg Northoff, Yu-Feng Zang

**Corresponding authors,**

Georg Northoff, georg.northoff@theroyal.ca

Yu-Feng Zang, zangyf@hznu.edu.cn

**Replication of behavioral results.**

The behavioral performance during visual and auditory attention tasks (CRT tasks) was characterized with the measures of the mean of RT, the amplitude of RT-fluctuation (RT-PerAF in 0.01 to 0.1 Hz), and error rate. These measures were analyzed for the testing dataset and replication dataset (Table S1). The replication dataset employed a slightly modified structure of paradigm, i.e. interleaving task and rest blocks (instead of having them separate in the testing dataset) (Fig. S1a). The block structure interleaves task and rest blocks (40 trials, 2-min for each). In rest blocks, participants were requested to fixate a cross in the middle of the screen. In the task block, the participants press the right/left button to indicate the right/left arrows (visual task) or the sine tones of 1000Hz/2000Hz (auditory task). Visual and auditory tasks were conducted in independent runs with counterbalanced order, while each run lasted about 12 min including 3 task blocks and 3 rest blocks. Then, the RT-PerAF in the frequency range of 0.01 to 0.1 Hz was calculated for each task block and was further averaged across all task blocks. Then, the averaged RT-PerAF was recruited in the subsequent analysis. We first determined that the RT-PerAF during the modified task (block paradigm of replication dataset) was stronger than that in the non-modified task (state paradigm of testing dataset) (modified task vs. non-modified task: visual modality, *t=*15.8, *p<*0.0001; auditory modality, *t=*6.07, *p<*0.001) (Table S1). Then, using Spearman rank correlation, we determine that the RT-PerAF has no significant correlation with the error rate of each modality (visual modality, *r=*0.28, *p=*0.08; auditory modality, *r=*0.18, *p=*0.25). Then, we reproduced the transmodal correlation of the RT-PerAF in the frequency range of 0.01 to 0.1Hz (*r=*0.44, corrected *p<*0.05) (Fig. S1b), and this correlation was not significant in the frequency range below 0.01 Hz (*r=*0.04, corrected *p>*0.05) and above 0.1 Hz (*r=*0.36, corrected *p>*0.05) after multiple corrections (Bonferroni correction for the correlations in the 3 frequency ranges). These replicated results strongly support the functional significance of slow RT-fluctuation (0.01 to 0.1 Hz) for sustained attention.

**
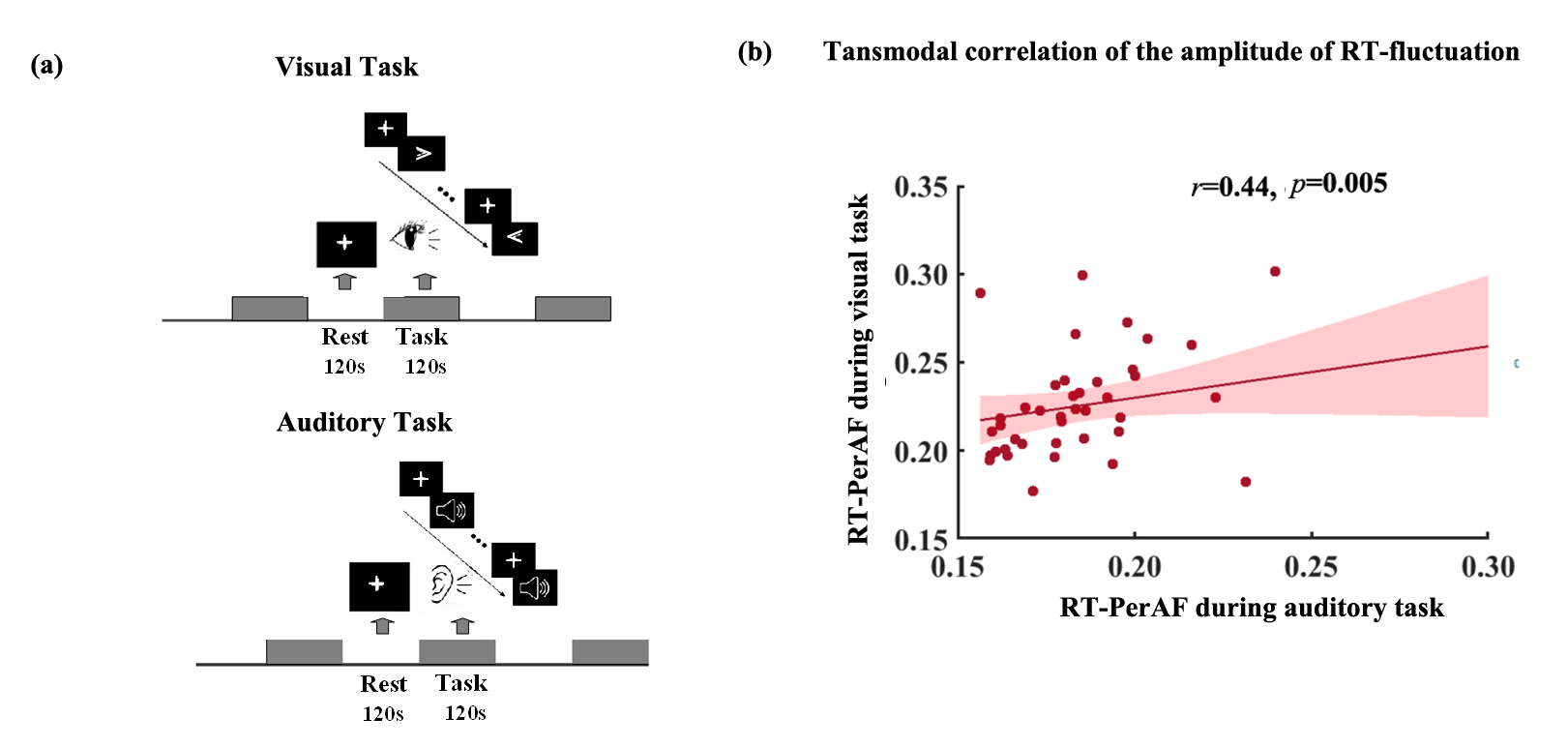
**

Fig. S1. Replication of transmodal correlation of visual and auditory RT-PerAF. The paradigm structure for the replication dataset (a); the transmodal correlation of the RT-PerAF in the frequency range of 0.01 to 0.1 Hz (b). Two outliers (individual’s value > 3 standard deviation from the group mean) were removed from the correlation analysis.

Table S1. The behavioral performance during visual and auditory tasks

| **Task type** | **Mean of RT (ms)** | **RT-fluctuation**  **(0.01-0.1 Hz)** | **Error Rate** |
| --- | --- | --- | --- |
| **Main testing dataset** | | | |
| **Visual Modality** | 449±61 | 0.09±0.03 | 0.04±0.02 |
| **Auditory Modality** | 631±174 | 0.16±0.06 | 0.07±0.03 |
| **Replication dataset** | | | |
| **Visual Modality** | 467±87 | 0.18±0.03 | 0.04±0.03 |
| **Auditory Modality** | 611±100 | 0.22±0.03 | 0.04±0.02 |

**Activation, deactivation, and fluctuation in sustained attention.**

The block paradigm allows us simultaneously measure activation, deactivation, and task-related fluctuation. We first determined regional loci showing activation, deactivation, and task-related fluctuation during the visual/auditory task (corrected with FDR q<0.05, cluster >10 voxels). Then, the transmodal overlaps of these measures were assessed with dice’s coefficient (Nei and Li 1979). We observed that regions of supplementary motor area (SMA), middle frontal gyrus (mFG), pre-motor area (PMA), posterior parietal lobule (PPL), insula (Ins.) thalamus (Thal.), basal ganglia (BG), and cerebellum (Cerebe,) showed transmodal activation (Table S2). Task-related fluctuation in the regions of superior frontal gyrus (sFG), ventral medial prefrontal cortex (vmPFC), posterior cingulate cortex (PCC), and angular had transmodal nature (Table S3). As Fig.S2a shows, the activation and task-related fluctuation were spatially distinct; that is activation in AN and fluctuation in DMN, and very few overlapped voxels (11 voxels) were identified in the region of the superior frontal gyrus/supplementary motor area.

Furthermore, we probed deactivation during visual and auditory tasks. Regions of PCC/precuneus, vmPFC, sFG, occipital lobe, and fusiform were deactivated in both tasks (Table S4 and Fig.S3a-c). These regions are mainly located in the mask of DMN (Fig.S3c), and have overlaps with the task-related fluctuation in the core regions of DMN, i.e. PCC and vmPFC (Fig. S2b).

Together, these findings suggested that the activation and task-related fluctuation could be dissociated, i.e. activation (AN) and task-related fluctuation (DMN). Deactivated DMN has another transmodal way, i.e. fluctuation, to support sustained attention.

**
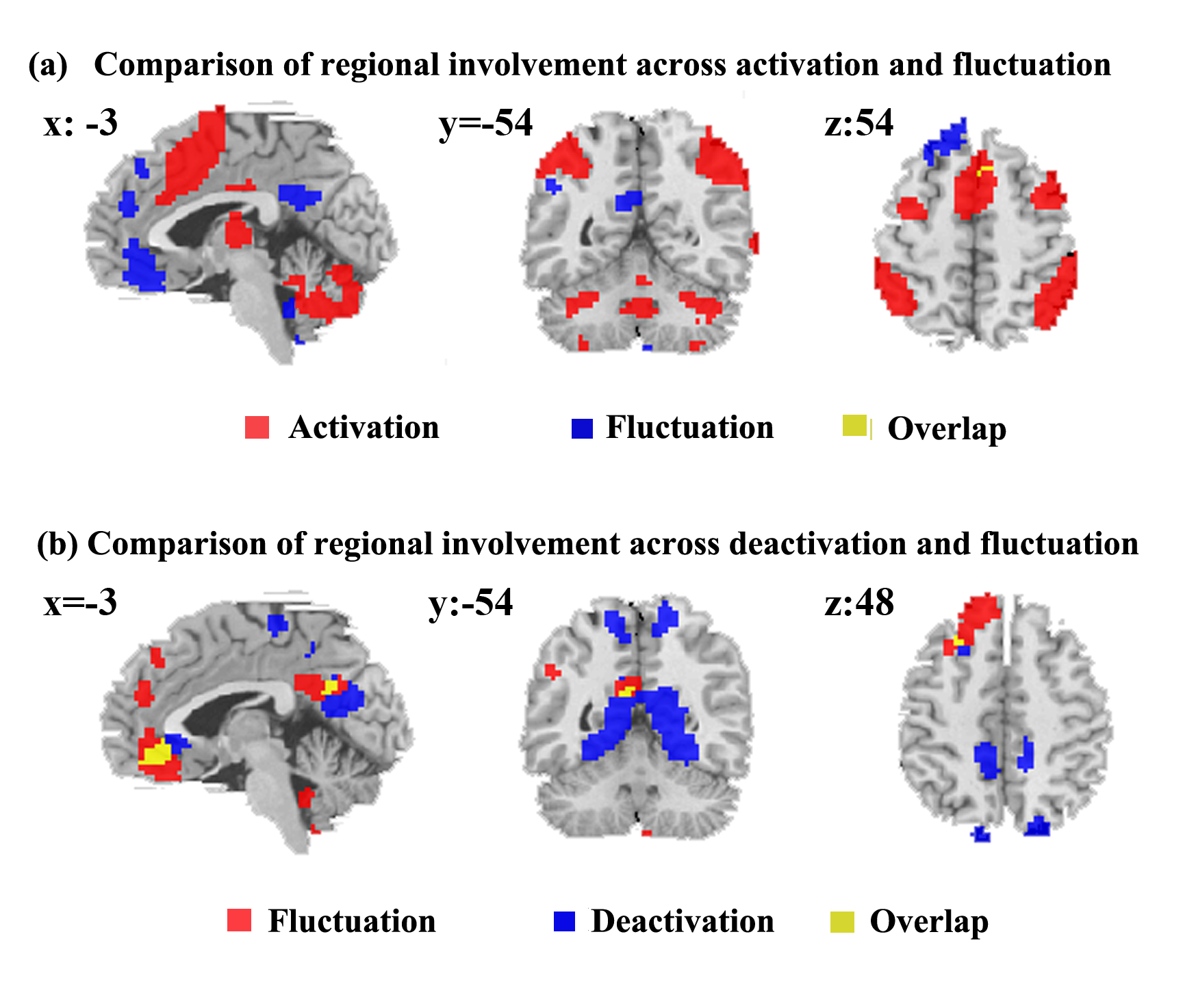
**

Fig. S2. Comparison of the transmodal involvement of regional loci across the measures of activation, deactivation and task-related fluctuation. The similarity and difference of regional involvement across (a) activation and fluctuation as well as (b) deactivation and fluctuation were determined respectively.


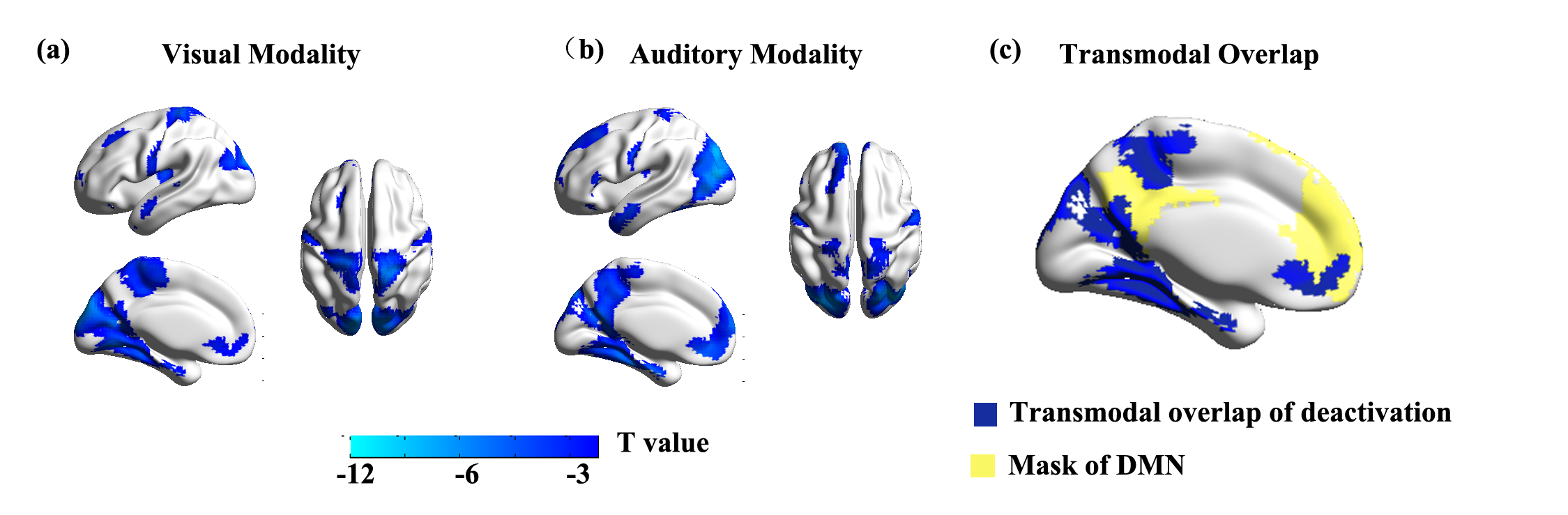


Fig. S3. Transmodal deactivation during sustained attention. Deactivation during visual task(a), auditory task (b) and their transmodal overlap (c). The overlapping regions are mainly located in the mask of default mode network. The network mask were released by Yeo et al., 2011, Deactivation was assessed using GLM to generate task-rest contrasts of the beta value, and this was multiple corrected with FDR, q<0.05, cluster size>10 voxels.

Table S2 Regions showing transmodal overlap of activation

| **Region** | **R/L** | **BA** | **Dice Coefficient** | **Visual Modality** | | | | | **Auditory Modality** | | | | |
| --- | --- | --- | --- | --- | --- | --- | --- | --- | --- | --- | --- | --- | --- |
|  |  |  |  | **x** | **y** | **z** | **T_peak_** | **Number of voxels** | **x** | **y** | **Z** | **T_peak_** | **Number of voxels** |
| SMA | R&L | 6 | 0.85 | -3 | -6 | 51 | 11.3 | 504 | 0 | 18 | 45 | 10.7 | 641 |
| mFG | R | 8 | 0.51 | 45 | 27 | 42 | 5.44 | 358 | 45 | 33 | 30 | 6.25 | 413 |
| PMA | R | 6 | 0.71 | 39 | 3 | 57 | 6.27 | 144 | 48 | 6 | 48 | 6.01 | 103 |
| PMA | L | 6 | 0.35 | -33 | -3 | 54 | 4.81 | 77 | -45 | 0 | 51 | 5.87 | 229 |
| PPL | R | 7/40 | 0.84 | 48 | -51 | 54 | 6.89 | 736 | 51 | -45 | 51 | 8.09 | 698 |
| PPL | L | 7/40 | 0.71 | -39 | -54 | 54 | 4.92 | 640 | -36 | -48 | 42 | 5.29 | 682 |
| Ins. | R | 13/47 | 0.61 | 48 | 21 | -3 | 6.01 | 90 | 48 | 15 | -3 | 10.5 | 207 |
| Ins. | L | 13/47 | 0.54 | -45 | 6 | 0 | 5.25 | 90 | -48 | 3 | 3 | 8.95 | 246 |
| Thal. | R&L | / | 0.93 | 6 | -21 | 9 | 5.80 | 397 | 3 | -6 | 6 | 7.07 | 345 |
| BG | R | / | 0.57 | -24 | 9 | -3 | 4.61 | 168 | 21 | 3 | 0 | 4.93 | 217 |
| BG | L | / | 0.90 | 21 | 12 | 0 | 3.97 | 163 | -21 | 6 | -3 | 4.99 | 195 |
| Cerebe | R | / | 0.78 | 36 | -63 | -30 | 7.9 | 337 | 27 | -63 | -30 | 6.04 | 238 |
| Cerebe | L | / | 0.84 | -36 | -63 | -30 | 6.87 | 367 | -33 | -66 | -30 | 7.73 | 306 |

Abbrevions:R, right hemisphere, L, left hemisphere, BA, brodmann area, MNI coordinate,

Table S3 Regions showing transmodal overlap of task-related fluctuation.

| **Regions** | **R/L** | **BA** | **Dice Coefficient** | **Visual task** | | | | | **Auditory task** | | | | |
| --- | --- | --- | --- | --- | --- | --- | --- | --- | --- | --- | --- | --- | --- |
|  |  |  |  | **x** | **y** | **z** | **T_peak_** | **Number of voxels** | **x** | **y** | **z** | **T_peak_** | **Number of voxels** |
| sFG | R | 8 | 0.62 | 9 | 18 | 54 | 3.65 | 559 | 12 | 30 | 60 | 4.45 | 667 |
| sFG | L | 8/9/`10 | 0.07 | -12 | 39 | 51 | 4.53 | 46 | -15 | 27 | 57 | 5.35 | 328 |
| vmPFC | L/R | 11/32 | 0.54 | -3 | 39 | -12 | 5.12 | 316 | 3 | 48 | -3 | 4.62 | 396 |
| PCC | L | 31 | 0.46 | -3 | -45 | 27 | 4.1 | 76 | -6 | -48 | 27 | 5.05 | 227 |
| Angular | R | 7/40 | 0.26 | -48 | -57 | 33 | 3.52 | 46 | -48 | -69 | 33 | 5.92 | 212 |

Abbrevions:R, right hemisphere, L, left hemisphere, BA, brodmann area, MNI coordinate,

Table S4 Regions showing transmodal overlap of deactivation.

| **Regions** | **R/L** | **BA** | **Dice Coefficient** | **Visual task** | | | | | **Auditory task** | | | | |
| --- | --- | --- | --- | --- | --- | --- | --- | --- | --- | --- | --- | --- | --- |
|  |  |  |  | **x** | **y** | **z** | **T_peak_** | **Number of voxels** | **x** | **y** | **z** | **T_peak_** | **Number of voxels** |
| sFG | L | 8 | 0.16 | -24 | 27 | 45 | -4.64 | 91 | -12 | 57 | 39 | -7.41 | 385 |
| vmPFC | L/R | 11/32 | 0.18 | 6 | 39 | -12 | -4.12 | 134 | -12 | 39 | -6 | -6.6 | 515 |
| PCC/  Precueus | L/R | 30/7 | 0.28 | -6 | -60 | 55 | -5.66 | 686 | -12 | -51 | 6 | -6.76 | 1133 |
| Occipital lobe | L | 19 | 0.32 | -12 | -93 | 30 | -9.1 | 535 | -24 | -84 | 33 | -9.34 | 955 |
| Occipital lobe | R | 19 | 0.37 | 21 | -93 | 36 | -9.75 | 513 | 30 | -81 | 36 | -9.88 | 843 |
| Fusiform | L | 20/37 | 0.35 | -18 | -63 | -9 | -7.31 | 218 | -27 | -45 | -12 | -6.68 | 366 |
| Fusiform | R | 20/37 | 0.39 | 24 | -51 | -12 | -7.46 | 271 | 30 | -51 | -12 | -6.93 | 396 |

Abbrevions:R, right hemisphere, L, left hemisphere, BA, brodmann area, MNI coordinate,

**Frequency-specificity of brain-behavior relationship**

To determine the frequency specificity of the brain-behavior relationship, we correlate the DMN-PerAF with RT-PerAF again in the frequency range below 0.01 Hz and above 0.1 Hz (testing dataset). No significant correlations during visual/auditory task were found in these frequency ranges (Fig. S4); these confirmed the specificity of the brain-behavior relationship in the frequency range of 0.01 to 0.1Hz

**
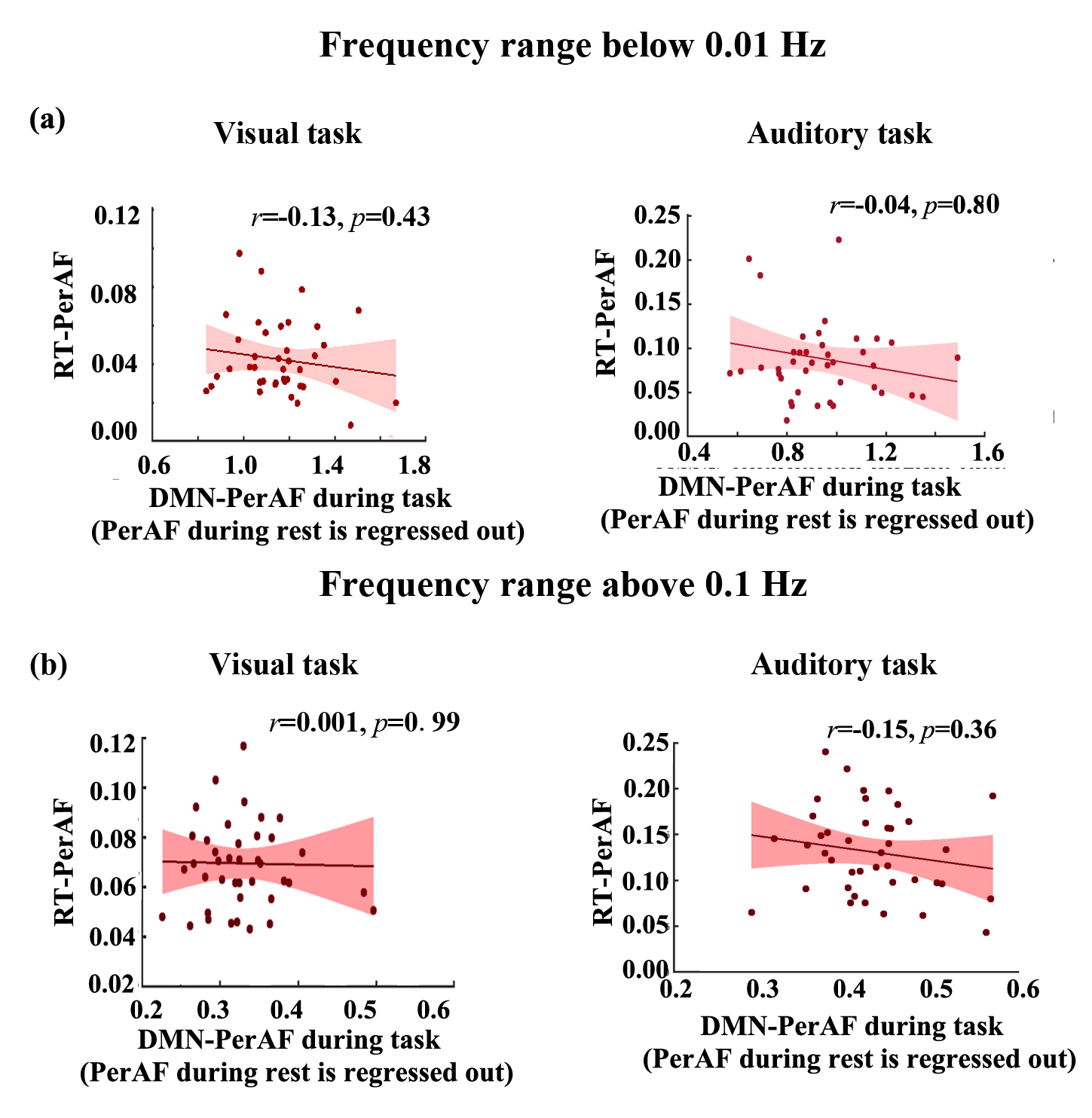
**

Figure S4. Validation of brain-behaviour relationship by correlating the DMN-PerAF with RT-PerAF in different frequency ranges. The correlations were assessed during both visual and auditory tasks(testing dataset), and were analyzed in the frequency ranges below 0.01 Hz (a) and above 0.1 Hz (b) respectively. Outliers (individual’s value> 3 standard deviation from the group mean) were removed from the correlation analysis.

**Replication of the brain-behaviour relationship**

Using the replication dataset, we again observed that the RT-PerAF was correlated with the DMN-PerAF during task but not during rest (Fig. S5); this confirmed that the neural fluctuation in DMN mediates the RT-fluctuation during sustained attention in the slow-frequency range.

**
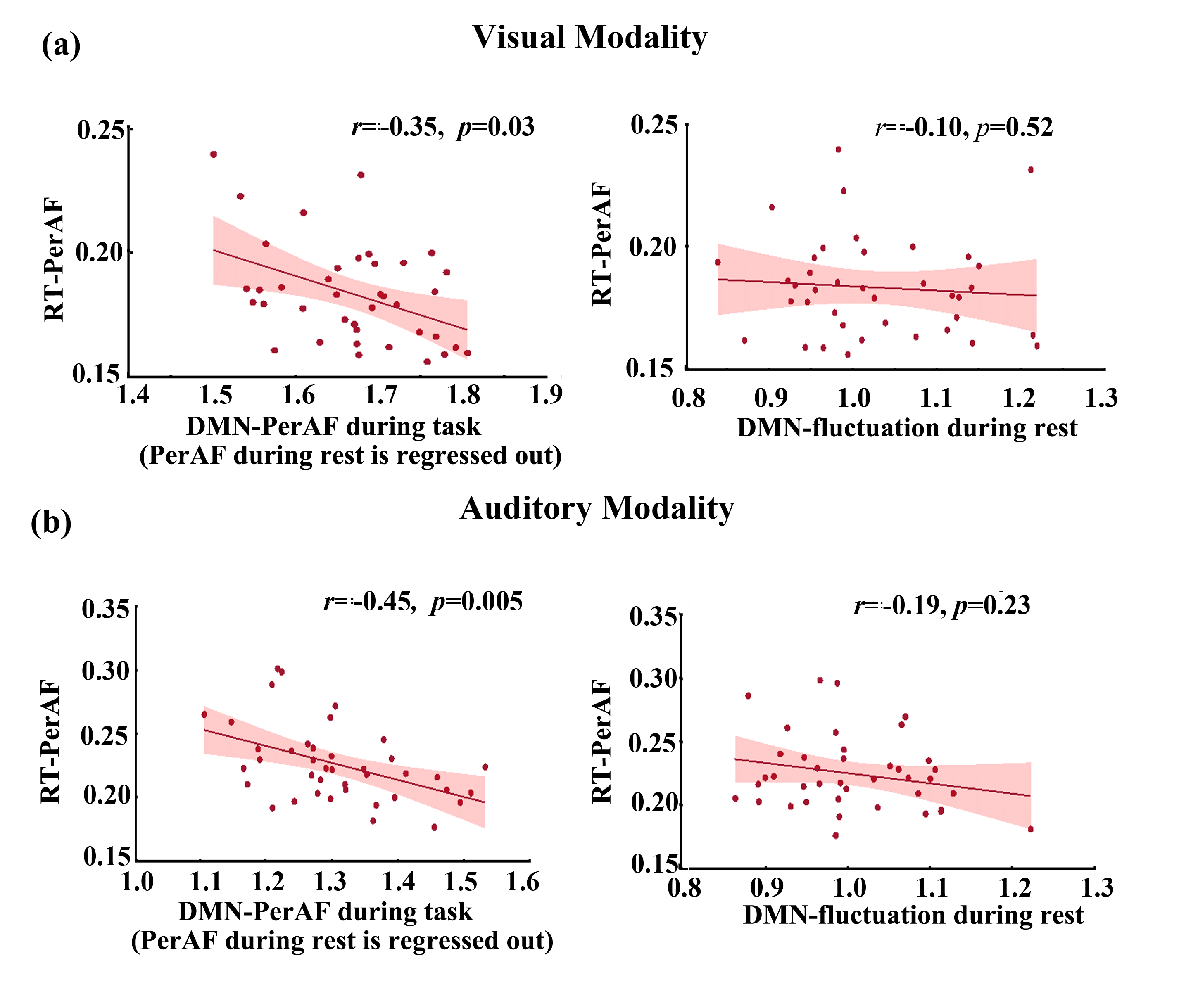
**

Fig. S5. Replication of the brain-behaviour correlation. The correlation between the DMN-PerAF and the RT-PerAF was again determined during the visual task (a) and auditory task(b). This correlation did not exist during rest. All correlations were assessed using Spearman rank correlation analysis. Outliers (individual’s value > 3 standard deviation from the group mean) were removed from the analysis.

**FC-fluctuation analysis**

In contrast to the PerAF measuring intra-regional fluctuation, we investigate FC-fluctuation for assessing inter-regional fluctuation. Both intra-network and inter-network fluctuation of FC were explored. The intra-network fluctuation was first determined from each pair of the sub-regions of AN/DMN using the dynamic FC analysis (sliding window, window size of 60s, and window step of 2s). The averaged fluctuation of intra-network FC across all pairs of sub-regions was then compared between AN and DMN, and we did found any significant difference (Fig.S6a and S6b). These observations were replicated with the window size of 30s and 120s (Fig.S6c and S6d). Inter-network fluctuation was evaluated for DMN and DAN using the window size of 60s and window step of 2s. As Fig.S7 shows, the fluctuation of inter-network FC has no difference between visual and auditory modalities, and these results could be replicated with the window size of 30s and 120s. We determined that intra- and inter-network fluctuations of FC have no correlation with the amplitude of RT-fluctuation; this further highlighted that the DMN mediates RT-fluctuation via its intra-regional fluctuation but not via inter-regional fluctuation. .

**
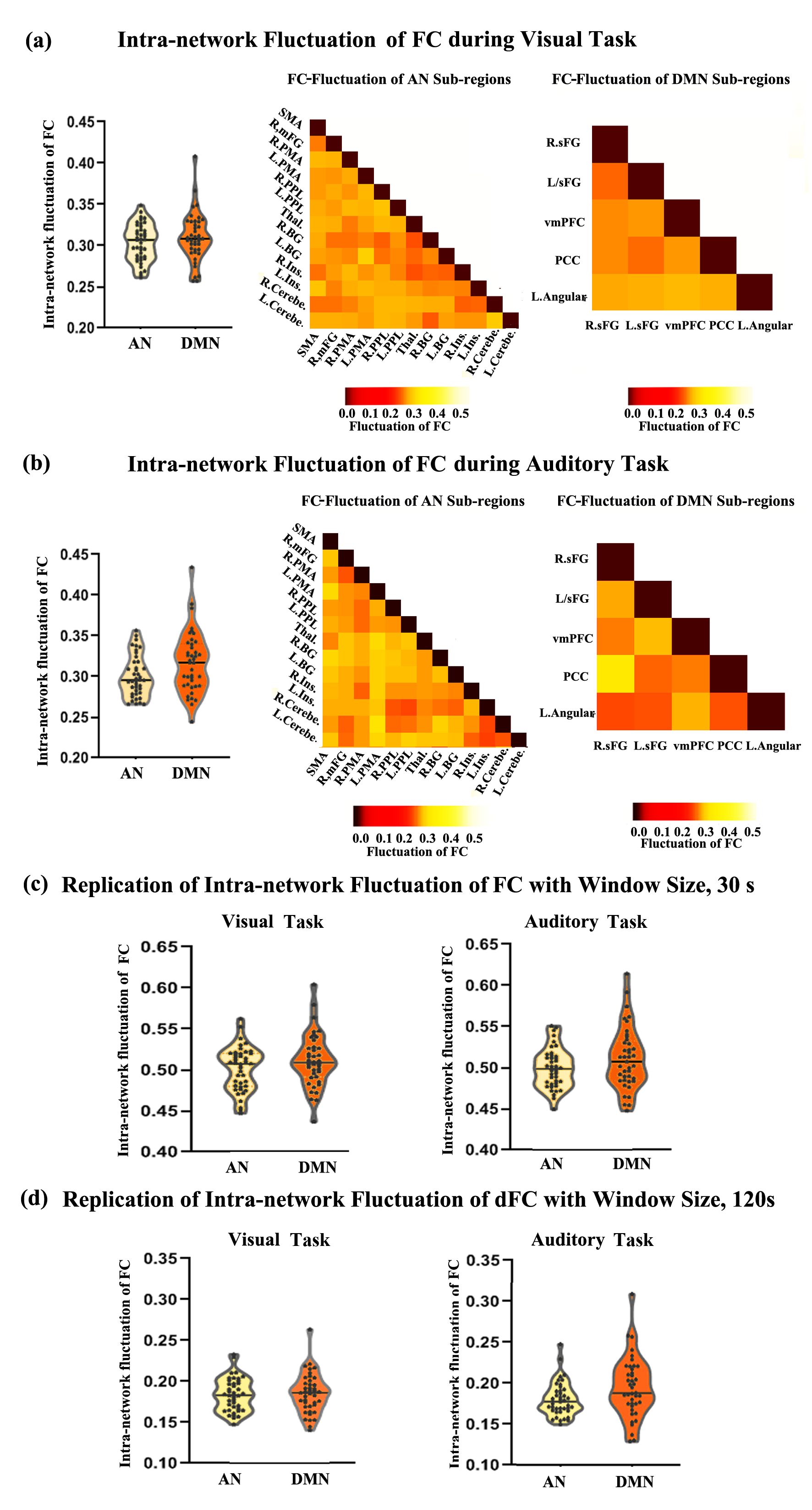
**

Fig. S6. Intra-network fluctuation of FC during visual and auditory tasks. Fluctuation of FC for each pair of the sub-regions of AN/DMN was assessed in visual task (a) and auditory task (b). The averaged fluctuation of intra-network FC across all pairs of sub-regions did not show significant difference between the AN and DMN. Each *|t|*<2.52 could not be survived under Bonferroni correction, *p<*0.05. These results were replicated with the window size of 30s (c) and 120s (d).

Table S5. The intra-/inter-network fluctuations of FC and their correlation with RT-fluctuations in visual and auditory modalities.

| **Measures of FC** | **Visual task** | | | **Auditory task** | | |
| --- | --- | --- | --- | --- | --- | --- |
|  | **Value of measures** | **Correlation with RT-fluctuation** | | **Value of measures** | **Correlation with RT-fluctuation** | |
|  | **Mean±SD** | ***r*** | ***p*** | **Mean±SD** | ***r*** | ***p*** |
| Intra-DMN fluctuation of FC (window size, 30s) | 0.51±0.03 | -0.02 | 0.88 | 0.51±0.04 | -0.05 | 0.76 |
| Intra-DMN fluctuation of FC (window size, 60s) | 0.31±0.03 | 0.05 | 0.76 | 0.32±0.04 | 0.08 | 0.60 |
| Intra-DMN fluctuation of FC (window size, 120s) | 0.19±0.02 | 0.17 | 0.29 | 0.19±0.04 | 0.12 | 0.47 |
| Intra-AN fluctuation of FC  (window size, 30s) | 0.50±0.03 | -0.03 | 0.86 | 0.50±0.02 | -0.05 | 0.77 |
| Intra-AN fluctuation of FC  (window size, 60s) | 0.30±0.02 | 0.05 | 0.76 | 0.30±0.03 | 0.03 | 0.83 |
| Intra-AN fluctuation of FC  (window size, 120s) | 0.18±0.02 | 0.24 | 0.14 | 0.18±0.02 | -0.07 | 0.68 |
| Inter-network fluctuation of FC  (window size, 30s) | 0.52±0.07 | -0.25 | 0.12 | 0.55±0.09 | -0.10 | 0.52 |
| Inter-network fluctuation of FC  (window size, 60s) | 0.32±0.07 | 0.07 | 0.65 | 0.35±0.08 | -0.21 | 0.18 |
| Inter-network fluctuation of FC  (window size, 120s) | 0.19±0.07 | 0.28 | 0.08 | 0.22±0.07 | -0.25 | 0.12 |
| Inter-network strength of FC | 0.35±0.28 | 0.20 | 0.20 | 0.39±0.26 | 0.19 | 0.24 |
